# The Impact of Inflammation on the Etiopathogenesis of Benign Salivary Gland Tumors: A Scoping Review

**DOI:** 10.3390/ijms252312558

**Published:** 2024-11-22

**Authors:** Konrad Szydłowski, Michał Puchalski, Stanisław Ołdziej, Agnieszka Kasprzyk-Tryk, Andrzej Skorek, Dmitry Tretiakow

**Affiliations:** 1 Department of Otolaryngology, Academy of Applied Medical and Social Sciences, 82-300 Elblag, Poland; ag.kasprzyk@wp.pl (A.K.-T.); askorek@copernicus.gda.pl (A.S.); d.trietiakow@gmail.com (D.T.); 2 The Nicolaus Copernicus Hospital in Gdansk, Copernicus Healthcare Entity, Powstańców Warszawskich 1/2, 80-152 Gdansk, Poland; 3 Intercollegiate Faculty of Biotechnology UG&MUG, University of Gdańsk, Abrahama 58, 80-307 Gdańsk, Poland; michal.puchalski@phdstud.ug.edu.pl (M.P.); stanislaw.oldziej@biotech.ug.edu.pl (S.O.)

**Keywords:** biomarkers, diagnosis, parotid gland, submandibular gland, parotidectomy, saliva, Warthin’s tumor, pleomorphic adenoma, parotid neoplasms

## Abstract

Salivary gland tumors are rare head and neck tumors constituting up to 6% of all head and neck neoplasms; despite being mostly benign, these tumors present in diverse histological subtypes, making them challenging to diagnose and treat. Our research aims to investigate the link between inflammation and tumorigenesis within the salivary glands based on the literature regarding the etiopathogenesis of benign salivary gland tumors. This scoping review was conducted following the PRISMA extension for scoping reviews and reporting guidelines. The search was conducted using the Pubmed and Embase databases. Articles published between 2004 and May 2024 were included in the review. A total of 1097 papers were collected and identified. After 271 duplicates were removed, 826 titles and abstracts were independently reviewed by two researchers. Based on the title and abstract, 735 citations were excluded, and 91 articles were assessed for eligibility. Data were extracted from 46 articles that met the inclusion criteria. The review highlights the significance of inflammation-related factors and its relations with benign salivary gland tumors (SGTs). Knowledge of the etiopathogenesis of these tumors remains insufficient, and their rich immunological background poses challenges in diagnosis. The findings also point to directions for further clinical research, which will be necessary to implement these molecules in clinical practice.

## 1. Introduction

Salivary gland tumors (SGTs) are diverse tumors that can be benign or malignant and mainly originate in the parotid salivary glands. SGTs are rare head and neck tumors constituting up to 6% of all head and neck neoplasms. Overall, almost 80% of tumors originate in parotid glands, of which 75% are benign and 25% are malignant [1]. According to the 2022 WHO classification, there are fifteen benign and twenty-one malignant epithelial neoplasms, one benign mesenchymal tumor, and two non-neoplastic epithelial lesions [2]. Due to their wide variety and complex histological composition, these tumors present in diverse histopathological subtypes, making them challenging to diagnose and treat [2]. The two most common parotid gland tumors are pleomorphic adenoma (PA), also known as a mixed tumor, and Warthin’s tumor (WT), also known as papillary cystadenoma lymphomatosum. These tumors account for almost 93% of all benign neoplasms originating in parotid glands [3,4]. Although benign, both of these tumors have malignancy potential, significantly higher in WT (up to 15%) than in PA (1%) [1,4]. The etiopathogenesis of benign parotid gland tumors is not clearly understood. Still, there are several risk factors, including chronic alcohol intake, age, and tobacco smoking [5,6]. Diagnosis using fine-needle aspiration biopsy has an average sensitivity of about 60 to 90% for lesions in the salivary glands, including differentiating between benign and malignant tumors [7,8,9,10]. Our research aims to investigate the link between inflammation and tumorigenesis within the salivary glands based on the literature regarding the etiopathogenesis of benign salivary gland tumors. The implementation of new biomarkers for the diagnosis of these tumors could increase their sensitivity and also allow for further development toward targeted biological treatment as an alternative to surgical intervention, which potentially carries the risk of damaging the facial nerve.

### Chronic Inflammation and Tumorigenesis

Chronic inflammation has been linked to neoplasm formation since the 19th century, firstly by the German pathologist Rudolf Virchow, who described inflammatory factors inside the tumor tissue [11]. Recently, many studies have shown a link between chronic inflammation and various stages of tumorigenesis [12,13,14,15]. Chronic inflammation may not only be a predisposing factor of tumorigenesis due to a pre-existing inflammatory state but is also responsible for the development and growth within the tumor itself as a tumor-induced inflammation. Inflammation, whether parallel to chronic inflammatory disease or in connection with tumor-induced inflammation, significantly affects the structure of the tumor microenvironment (TME) and the adaptability of both the tumor and surrounding cells. Growing tumors create a continuous cycle of inflammation-induced signaling, facilitating the recruitment of inflammatory molecules. This contrasts with the regular inflammatory pathway, which resolves after limiting the initiating factor. One of the most critical steps in tumorigenesis is tumor initiation. The inflammatory response has the potential to accumulate gene mutations and epigenetic modifications. These changes can deactivate the immune system to suppress tumors and activate pathways that promote cancer. Inflammation unequivocally increases mutagenesis and predisposes normal tissue to accumulate mutations. Another essential part of the development of neoplasms is tumor promotion. Again, the inflammatory process plays a significant role, as research shows that inhibiting inflammation can alter tumor growth. As in tumor initiation, inflammatory cells and their signaling molecules can act as growth factors for growing tumors. Moreover, inflammatory factors play a crucial role in shaping cell plasticity within the tumor microenvironment, ultimately influencing the growth of tumors [13]. The tumor microenvironment is the specific cellular environment in which a tumor exists, consisting of different immune factors, signaling molecules, and extracellular matrix components. It is believed it plays a crucial role in the pathogenesis of tumor growth and its ability to develop. Numerous pathways and molecules, including pro-inflammatory cytokines and chemokines, can trigger and sustain tumorigenesis, creating an environment that helps neoplastic cells grow and thrive [16,17,18].

Additionally, chronic inflammation can have a significant impact on the development of cancer, affecting everything from tumor initiation and promotion to progression and metastasis [12].

## 2. Methods

This scoping review was conducted following the Preferred Reporting Items for Systematic Reviews and Meta-Analyses (PRISMA) extension for scoping reviews and reporting guidelines [19].

### 2.1. Search Strategy

The search was conducted using Pubmed and Embase databases. Articles published between 2004 and May 2024 were included in the review. The search terms used were ”salivary gland neoplasms” and “salivary gland tumors”, combined with terms including “benign”, “inflammation”, and “pathogenesis”.

### 2.2. Inclusion and Exclusion Criteria

Studies investigating salivary gland tumors involving benign tumors were included. The search was restricted to articles written in English. Only human studies were included in the review. Articles analyzing chromosomal translocations, genetic disturbances, and infectious diseases as etiology of benign salivary gland tumors were excluded.

### 2.3. Data Extraction

After duplicates were removed using Mendeley’s reference management software 2.110.2, two authors (K.S. and D.T.) independently reviewed articles. The titles and abstracts of selected articles were screened, and those articles underwent evaluation for eligibility.

## 3. Results

**Figure 1:**
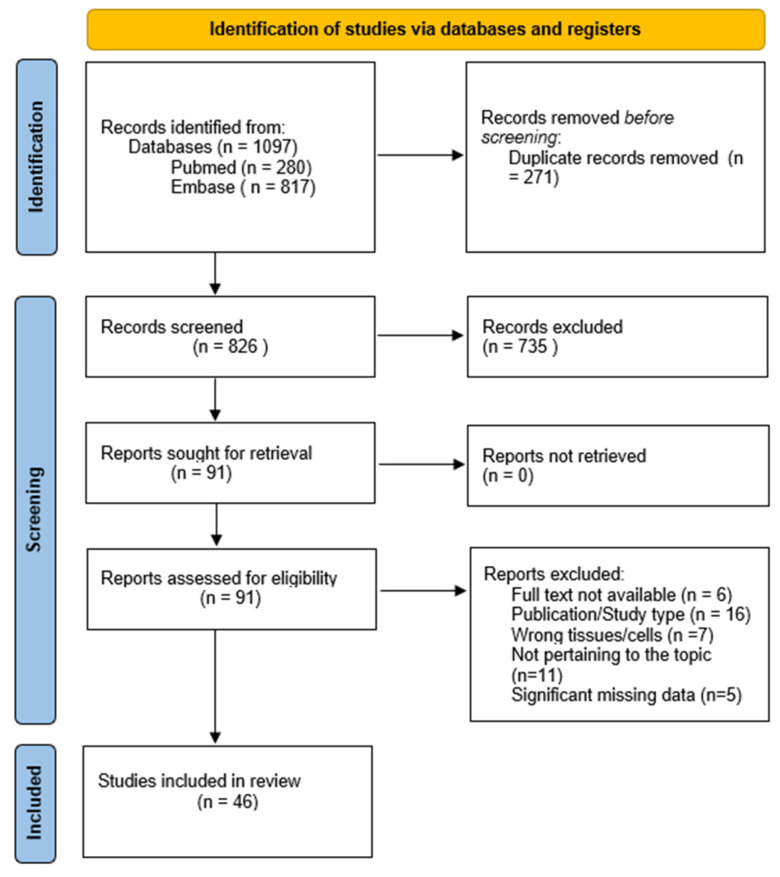
PRISMA flow diagram.

As shown in Figure 1, 1097 papers were collected and identified. After 271 duplicates were removed, 826 titles and abstracts were independently reviewed by two researchers. Based on the title and abstract, 735 citations were excluded, and 91 articles were assessed for eligibility. Data were extracted from 46 articles (Table 1) that met the inclusion criteria.

**Figure 2:**
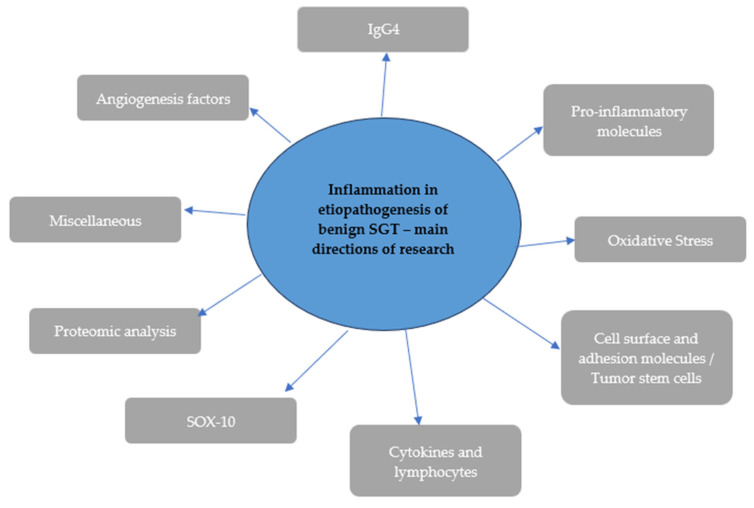
Main directions of research.

The following main research directions were observed: angiogenesis factors, IgG4, pro-inflammatory molecules, oxidative stress, cell surface, and adhesion molecules, tumor stem cells, cytokines and lymphocytes, SOX-10, proteomic analysis, and others (Figure 2). Most of the analyzed studies were retrospective, randomized, observational studies using healthy tissues or tissues obtained from patients with malignant tumors as control groups. The review also included several case series studies. Of the forty-six studies included in the literature review, eight investigated substance concentrations in plasma, two involved both plasma and tumor tissues, and the remaining studies focused on the expression of molecules in removed tumor tissues or the impact of these molecules on tumor development. The main method for determining markers among selected articles was immunohistochemistry. In some of the studies, molecular research also included the assessment of mRNA expression.

## 4. Discussion

### 4.1. Angiogenesis Factors

Among the selected group of studies, the most commonly investigated factor was VEGF or its receptors. These molecules play a crucial role in stimulating the process of angiogenesis, which is key to tumor development and also plays an important role in inflammation [66,67]. Most studies analyzing the role of VEGF reported its overexpression in tumor tissues and elevated levels in the plasma of patients with salivary gland tumors [23,28,36,63]. In the study by Jabbar et al., VEGF overexpression was detected in 29 out of 30 benign salivary gland tumors [63]. Similarly, Faur et al. found an increased VEGF expression and mean microvascular density (MVD) in benign tumors compared to the control group, although these values were generally lower than in malignant tumors [36]. However, Błochowiak et al. found no differences in VEGF expression or its receptors between benign tumors and healthy tissue surrounding the tumor (control group) [49]. These results may be due to the study’s focus on specific isoforms, such as VEGF165 and VEGF165b, which were analyzed. Tampouris et al. demonstrated that pleomorphic adenomas had the strongest predilection for the VEGF C/D–VEGFR3 (flt-4) axis compared to other benign and malignant salivary gland tumors [23]. Tadbir et al. analyzed VEGF levels in the plasma of patients with salivary gland tumors [28]. The average plasma VEGF concentration was the highest in the malignant tumor group (727.3 ± 441.8 pg/mL), slightly lower in the benign tumor group (442.2 ± 343.3 pg/mL), and the lowest in the control group (263.9 ± 310.0 pg/mL) [28]. Gaonkar et al. investigated the expression of endoglin as an angiogenesis marker, showing its increased expression in both benign and malignant tumors compared to the control group [60]. The authors suggest that endoglin concentration in tissue may correlate with the degree of tumor malignancy [60]. The role of endoglin in inflammation, including its involvement in leukocyte adhesion and the formation of new blood vessels, has also been described [68,69]. Other factors involved in regulating tumor angiogenesis include semaphorins (acting as VEGF antagonists) and neuropilins, which function as VEGF coreceptors [42]. However, in a study of 248 benign and malignant tumors, no statistical differences were found in the expression of these molecules, limiting their use in diagnostics [42].

### 4.2. IgG4

Several studies focused on the presence of IgG4 in plasma or detecting these globulins in Warthin’s tumors and lymphadenomas [29,35,44]. Aga et al. described a case of a patient with Warthin’s tumor and IgG4-related autoimmune pancreatitis [29]. In addition to elevated IgG4 levels in the blood, immunoglobulins were also present in the tumor stroma [29]. In another study, Aga et al. evaluated blood plasma and tissue samples from patients with benign salivary gland tumors for the presence of IgG4, comparing their levels between patients with Warthin’s tumor and pleomorphic adenomas [35]. Elevated IgG4 levels were found in the plasma of five out of eighteen patients with Warthin’s tumor, and, in four of these, IgG4 was also present in tumor tissue, while no IgG4 was detected in the plasma or tumor tissue of patients with pleomorphic adenoma [35]. The study also analyzed the mRNA expression of these immunoglobulins in salivary gland tumor tissues and in a patient with IgG4-related disease (IgG4RD). IgG4 mRNA overexpression was detected in two of three analyzed Warthin’s tumors, similar to IgG4RD, but was not found in pleomorphic adenomas [35]. Kim J et al. analyzed IgG4 levels in tissues from fifteen lymphoepithelial tumors, including twelve benign tumors, eight of which were lymphadenomas and four were lymphoepithelial cysts [44]. Elevated IgG4 levels were statistically significant in two out of eight lymphadenomas, while no correlation was found in other types of lesions [44].

### 4.3. Pro-Inflammatory Molecules

In a study by Loy et al., an increased expression of COX-2 was observed in all 21 analyzed patients with Warthin’s tumor (WT) [20]. The enzyme was present in the epithelial component of the tumor, excretory ducts, and healthy salivary tissue adjacent to the tumor, while no increased expression was found in the remaining healthy salivary gland parenchyma [20]. Tenorio et al. compared 62 benign and malignant salivary gland tumors for COX-2 expression in their tissues [50]. A strong COX-2 overexpression was observed in all mucoepidermoid carcinoma tissues, while, in benign tumors, a weak expression was observed in 39.5% of pleomorphic adenomas (PAs), and in 60.5%, it was not detected at all [50]. The difference in COX-2 expression between these studies may stem from the different types of benign tumors analyzed (WT vs. PA), suggesting a predilection towards inflammatory pathway via COX-2 in Warthin’s tumor development.

In three studies, researchers collected blood samples from patients undergoing surgery for salivary gland tumors and analyzed leukocyte, platelet, neutrophil, and monocyte counts to calculate indexes representing the intensity of inflammation [57,61,64]. Abbate et al. analyzed inflammatory biomarkers (NLR, PLR, SII) in 191 patients with benign salivary gland tumors compared to 90 control patients [61]. The NLR and SII indexes were significantly higher in patients with salivary gland tumors compared to the control group. In contrast, the PLR index was elevated only in patients with pleomorphic adenomas. Sahin et al. evaluated whether the SII index correlated with the malignancy of salivary gland tumors [57]. Comparing 185 patients with benign tumors to 52 with malignant tumors, a statistically significant difference in SII levels was found, with higher values in malignant tumors. However, no cutoff value was established for using this biomarker in clinical practice [57]. In another study, Abbate et al. examined the use of inflammatory biomarkers (SII, SIRI, PLR, and NLR) in diagnosing salivary gland tumors alongside cytological results from fine-needle aspiration biopsies [64]. SIRI proved to be the most diagnostically useful biomarker, with the combination of SIRI and FNAC results increasing diagnostic sensitivity to 82.8% compared to 59.6% with FNAC alone and 66.7% with SIRI alone [64]. These findings suggest a strong association between inflammation and salivary gland tumors, including benign tumors, and highlight the usefulness of inflammatory biomarkers in diagnosing these tumors.

### 4.4. Oxidative Stress

Two of the studies analyzed plasma concentrations of substances that serve as markers of oxidative stress [48,62]. In the study by Sowa et al., the results indicate that patients with salivary gland tumors, including benign ones, exhibit a low level of whole-body oxidative stress [48]. This is evidenced by decreased levels of Total Antioxidant Capacity of Blood Serum (FRAP) and thiol groups and increased levels of Advanced Oxidation Protein Products (AOPP). In another study by Sowa et al., which involved 61 patients with benign and malignant salivary gland tumors (52 of whom had benign tumors), elevated blood lipofuscin levels were observed in all patients with salivary gland tumors compared to the control group, regardless of tumor type [62]. Additionally, the study showed a decrease in the antioxidant enzyme Cu-Zn SOD, but only in patients with Warthin’s tumors, suggesting the involvement of this antioxidant enzyme in the pathogenesis of this type of tumor [62]. The findings indicate that oxidative stress, commonly linked to tumor development and chronic inflammation in the literature, is present in patients with salivary gland tumors, including benign ones [70,71,72].

### 4.5. Cell Surface and Adhesion Molecules/Tumor Stem Cells

Many of the studies focused on detecting adhesion molecules and various receptors and ligands, including CD group receptors and cadherins [21,25,32,33,38,54,58,59]. These molecules, in addition to helping identify cell types in immunophenotyping studies, play significant roles in signaling pathways, including tumorigenesis and inflammation development [73,74]. Tumor stem cells, identified using biomarkers, were also evaluated [32,54,58,59]. These cells can initiate tumorigenesis and may be responsible for treatment failures and tumor recurrence [75].

Certain molecules may act as markers of malignant transformation risk, such as CD166. Tadbir et al. showed increased expression of CD166 in both benign and malignant tumors, with higher levels observed in malignant tumors [38]. In another study, the expression of CD44 was analyzed in benign and malignant salivary gland tumors [59]. CD44 is widely studied in the context of various types of cancer due to its role in cell adhesion and migration and its use as a tumor stem cell marker [76,77,78]. In a study of 38 patients, including 13 with benign tumors, CD44 and CD44v6 isoforms were found to be upregulated in benign tumors compared to the control group, though their levels were slightly lower than in malignant tumors [59]. Ianez et al. compared the expression of CD24 and CD44 between 101 surgically removed pleomorphic adenomas and 20 fetal salivary gland tissues [32]. The study assessed whether these molecules could be markers for salivary gland cancer stem cells. CD24 and CD44 were detected in all pleomorphic adenomas and fetal salivary glands using immunohistochemistry, but no characteristic expression pattern was found within the pleomorphic adenomas. The expression pattern for both markers was consistent between fetal salivary glands and normal human salivary glands used as a control group. Molecular analysis using RT-PCR revealed increased CD44 expression only in pleomorphic adenomas, with no difference in CD24 expression compared to the control group. The authors concluded that CD24 and CD44 could not be used as specific cancer stem cell markers for prognostic or diagnostic purposes [32]. Haghshenas et al. performed a proteomic analysis of five benign and five malignant salivary gland tumors to investigate proteins associated with mesenchymal stem cells [58]. They found overexpression of several markers and receptors, including CD44, CD73, CD90, CD105, and CD166, present in both benign and malignant tumors. Notably, an increased expression of heat shock protein 70 (Hsp70), keratin, and type II cytoskeletal 7 (CK-7) was observed in benign compared to malignant tumors [58]. These findings confirm the distinct architecture and etiopathogenesis of benign and malignant tumors. Two studies focused on cadherin expression, molecules involved in cell adhesion whose role in tumorigenesis has been investigated for many benign and malignant tumors [21,25,73]. In a study of 110 benign and malignant salivary gland tumors, a strong E-cadherin expression was observed in all 54 benign tumors, while malignant tumors showed a loss of expression as their cytological malignancy increased [21]. A study evaluating N-cadherin expression in 18 benign and 31 malignant salivary gland tumors found no expression in the control group tissues, pleomorphic adenomas, or myoepitheliomas [25]. However, N-cadherin was present in the epithelium of one Warthin’s tumor and the germinal centers of lymphoid tissue in four Warthin’s tumors [25]. Additionally, the study demonstrated a correlation between increased N-cadherin expression and histological malignancy and neuroinvasion in malignant tumors [25], suggesting its potential as a diagnostic biomarker. Karbanova et al. found an increased expression of Prominin (CD133) in both benign and malignant salivary gland tumors [33]. This overexpression was also present in the peritumoral tissues and inflamed salivary glands, suggesting a possible link between chronic irritation and tumor development. The authors hypothesize that tumor growth leads to cell compression, disrupting blood flow and causing hypoxia, which results in inflammation [33]. ALDH-1, an enzyme used as a marker for tumor stem cells, was analyzed in 154 salivary gland tumors by Da Silva et al. [54]. They found increased expression of ALDH-1 in all 51 benign tumors and 85.6% of the 103 malignant tumors [54]. The results confirm the presence of tumor stem cells in salivary gland cancers, which may impact disease progression and prognosis. In some malignant tumors, ALDH-1 expression in the stroma correlated with shorter overall survival, suggesting a prognostic role for tumor stem cells in malignant tumors, though further research is needed to understand their role in benign tumors [54].

### 4.6. Cytokines and Lymphocytes

Numerous studies have analyzed cytokine concentrations and expression in both tumor tissues and plasma from patients with benign salivary gland tumors [24,26,40,43,45,46,47,55,56].

One of the chemokines studied was CCL28, which is involved in lymphocyte recruitment and plays a vital role in the immune response, particularly in the oral cavity. Liu G et al. analyzed 72 benign salivary gland tumors, including pleomorphic adenomas and adenolymphomas, for the presence of CCL28 in the tissues. They found decreased expression of CCL28 in tumors compared to healthy surrounding tissues, suggesting that reduced recruitment of anti-tumor factors could be a mechanism in the tumorigenesis of these neoplasms [24]. Biological drugs, specifically monoclonal antibodies against various pro-inflammatory factors, are a groundbreaking class of medications used in treating many inflammatory and autoimmune diseases [79,80]. Carlesimo M et al. presented cases of two patients with psoriasis treated with Adalimumab (an anti-TNF alpha antibody) who developed benign salivary gland tumors during the treatment [26]. One patient developed bilateral Warthin’s tumors, while the other was diagnosed with a pleomorphic adenoma of the parotid gland. Although the limited number of cases does not allow for a definitive cause-and-effect relationship, this raises the possibility of a link between TNF alpha activity and tumorigenesis in benign salivary gland tumors. The distribution of T regulatory cells (Tregs), IL-17-producing lymphocytes, and CTLA4+ lymphocytes was analyzed in the plasma of patients with nineteen benign and eight malignant salivary gland tumors and compared to a control group [40]. An increased proportion of Tregs and CTLA4+CD4+ lymphocytes was found in both benign and malignant tumor patients compared to the control group, with the ratio being significantly higher in the malignant tumor group (7.74 ± 1.1 for Tregs and 8.18 ± 1.77 for CTLA4+CD4+ cells in malignant tumors, vs. 4.38 ± 0.56 for Tregs and 3.83 ± 0.56 for CTLA4+CD4+ cells in benign tumors, and 2.34 ± 0.28 for Tregs and 2.22 ± 0.25 for CTLA4+CD4+ cells in controls). Meanwhile, the percentage of Th17 cells and the Th17/Treg ratio was lower in both benign and malignant tumor groups compared to controls (Th17 cells: 0.84 ± 0.14 in malignant tumors, 2.09 ± 0.31 in benign tumors, and 2.31 ± 0.23 in controls; Th17/Treg ratio: 0.12 ± 0.03 in malignant tumors, 0.48 ± 0.09 in benign tumors, and 1.26 ± 0.23 in controls). These differences in lymphocyte subpopulations may suggest their role in the pathogenesis of tumors, with benign tumors appearing as an intermediate stage between malignant tumors and normal salivary gland tissues [40].

In another study, Haghshenas et al. analyzed the concentrations of lymphocyte subtypes (Th1, Th2, Tc1, and Tc2) in the peripheral blood of patients with benign and malignant salivary gland tumors [45]. No difference was observed in the Th1 and Th2 lymphocyte percentages between the benign tumor group and controls. However, the Th1/Th2 ratio in the benign tumor group (8.19 ± 1.56) showed a statistically significant decline compared to controls (11.13 ± 1.60). Similarly, no differences were found in the Tc1 and Tc2 percentages, though the Tc1/Tc2 ratio was lower in the benign tumor group (13.79 ± 4.16) compared to controls (16.40 ± 2.70), although this difference was not statistically significant (*p* = 0.12). Khademi B et al. [43] analyzed cytokine levels, specifically IFN-gamma (a Th1 marker) and IL-4 (a Th2 marker), in 55 benign tumors, 14 malignant tumors, and a control group. No significant differences were found in these cytokines between groups, suggesting no role in the etiology of salivary gland tumors [43]. However, Zare R et al. found a slightly higher expression of IL-33 in benign pleomorphic adenomas compared to healthy salivary tissue, though the expression was lower than in malignant tumors [46]. These results indicate that IL-33 may have potential as a biomarker for salivary gland tumor diagnostics.

Adipocytokines are a group of bioactive proteins involved in metabolic processes [81]. Sowa et al. analyzed circulating adipocytokines in the plasma of salivary gland tumor patients [47]. They found increased levels of adiponectin and visfatin in all 51 patients with benign tumors, regardless of gender. Additionally, higher leptin levels were observed in men with benign salivary gland tumors but not in women, with no difference in IL-6 levels between male and female patients compared to the control group [47].

Kobayashi et al. evaluated the potential role of helper T cells and follicular helper T cells in tissues from 64 patients with Warthin’s tumors, dividing the tumors into solid (25 tumors) and cystic (39 tumors) subtypes based on imaging studies [56]. Cystic tumors showed atrophy of germinal centers compared to solid tumors, resulting in a decrease in follicular helper T cells in the germinal centers. Cystic tumors also demonstrated an increase in T-bet-positive lymphocytes in the epithelium compared to solid tumors. Furthermore, Th2-induced responses were suppressed in cystic tumors, with a dominance of Th1 responses, leading to chronic inflammation and more significant tumor tissue destruction compared to solid tumors [56]. These differences in lymphocyte concentrations and ratios suggest that each subtype presents a unique microenvironment and architecture, which may reflect slightly different pathogenesis and behavior [56].

Mochizuki et al. compared the expression of CXCL12, CXCL10, and CCL18 between 20 Warthin’s tumors and 20 pleomorphic adenomas using immunohistochemical analysis and RT-PCR [55]. All Warthin’s tumors were immunopositive for CXCL12 and CXCL10, with most also positive for CCL18, while nearly all pleomorphic adenomas showed negative results [55]. The findings suggest that these chemokines are linked to the lymphatic stroma of Warthin’s tumors, playing a role in their pathogenesis and highlighting their histological distinction from pleomorphic adenomas.

### 4.7. SOX-10

Ohtomo et al. analyzed a total of 90 benign and malignant salivary gland tumors for the expression of SOX-10 protein, a transcription factor involved in the differentiation of various cell types, which is also present in the healthy tissue of major salivary glands [30,82]. Among the 14 benign tumors analyzed significant overexpression of SOX-10 protein was found only in pleomorphic adenomas, with no overexpression observed in Warthin’s tumors or oncocytomas [30]. The results confirm these tumor types’ distinct pathogenesis and nature and highlight SOX-10 as a valuable diagnostic marker. In the study by Baneckova et al. SOX-10 and S1009 protein expression was evaluated in 89 benign tumors, including 74 oncocytomas, 15 pleomorphic adenomas (PA), and myoepitheliomas (ME) with oncocytic metaplasia. SOX-10 and S1009 expression was detected in all pleomorphic adenomas and myoepitheliomas, and in 11 of the 74 oncocytomas [53]. Further histopathological analysis based on these findings suggests that tumors with positive expression of these proteins, initially diagnosed as oncocytoma, were oncocytic variants of PA and ME, which may help differentiate these tumors and shed new light on their pathogenesis [53].

### 4.8. Proteomic Analysis

Donadio et al. performed proteomic analysis on fine needle biopsy material from 36 benign parotid gland tumors, including 22 pleomorphic adenomas and 14 Warthin’s tumors [31]. A total of 26 different proteins were detected. An analysis of the identified proteins using dedicated computational software revealed two distinct protein networks for pleomorphic adenomas and Warthin’s tumors [31]. Among the proteins with increased expression in pleomorphic adenoma tissues were ANXA1, ANXA4, ApoE, and CRYAB, which are directly linked to the process of tumorigenesis [31,83,84]. On the other hand, the protein network detected in Warthin’s tumors (e.g., IGHG1, IGKC, IGHA1, and S100A9) was associated with immunological and inflammatory diseases [85]. These detected differences in tumorigenesis pathways between these tumors and identifying specific proteins offer hope for their use as biomarkers and could lead to improved preoperative diagnostics.

### 4.9. Miscellaneous

Human beta-defensin is a protein described in the literature as involved in tumor pathogenesis, potentially functioning as a tumor suppressor or acting as a proto-oncogene depending on the protein isoform and cancer type [86]. In the analyzed study, the expression of three hBD (1, 2, 3) subtypes was compared between healthy salivary gland tissue, chronically inflamed salivary glands, pleomorphic adenoma tissues, and adjacent adenoma tissues [22]. Pantelis et al. demonstrated statistically reduced expression of hBD1, with no statistical difference for hBD 2/3 in all five analyzed pleomorphic adenomas compared to healthy salivary gland tissue [22]. However, hBD1 and hBD3 expression levels were significantly elevated in inflamed salivary glands and adjacent adenoma tissues compared to healthy salivary tissue. The results suggest an important role for hBD1 as a tumor suppressor in pleomorphic adenoma tumorigenesis.

Cyclin D1 and p16INK4A proteins are involved in cell proliferation via regulation dependent on the Rb protein, and disturbances in this regulation can lead to tumor development. Jour et al. analyzed p16 and cyclin D1 protein expression in 44 salivary gland tumors, including 14 benign tumors. The overexpression of both proteins was observed in nearly all benign and malignant tumors compared to healthy tissue, but no statistically significant differences were found between salivary gland tumor types. The results argue against using these factors as diagnostic markers for distinguishing malignancy in salivary gland tumors [27].

Maspin is a protease inhibitor protein that acts as a tumor suppressor by inhibiting tumorigenesis processes [87]. Its role has been analyzed in many cancers, where its expression has been associated with either better or worse patient prognosis depending on the tumor type [34]. Reshma et al. compared maspin expression between benign and malignant salivary gland tumors, demonstrating the increased expression of this protein in all benign salivary gland tumors, while it was present in only a little over half of malignant tumors [34]. This could indicate a less aggressive nature for some malignant tumors. The results suggest the potential use of maspin as a diagnostic or prognostic marker in salivary gland tumors, given the milder nature of tumors expressing this protein.

Lipids are a significant component of the tumor microenvironment and have a notable impact on the process of carcinogenesis [88]. He Q et al. analyzed the distribution pattern of phosphatidylcholine subtypes within Warthin’s tumors compared to normal salivary gland tissue using imaging mass spectrometry [37]. Five different phosphatidylcholines were detected in all analyzed Warthin’s tumors, with only PC (16:0/16:0) being significantly elevated in the lymphoid stroma region of the Warthin’s tumors, which may indicate a distinct metabolism in this tumor region. The authors suggest a possible connection between this localization of PC (16:0/16:0) and the role of inflammation in the etiopathogenesis of this tumor [37].

PRDM1 is a molecule involved in the modulation of immune cells, including B and T lymphocytes [89]. Wang et al. analyzed PRDM1 protein in 40 Warthin’s tumors (WT), comparing its expression between the epithelial component of the tumor, surrounding healthy tissues, germinal centers of WT, and palatine tonsil tissues with follicular hyperplasia, which served as a reference for the lymphoid stroma of the Warthin’s tumor. They detected an overexpression of PRDM1 protein in all analyzed tumors within the epithelial component and palatine tonsil tissues, but no expression was observed in the lymphoid germinal centers or tissues surrounding the tumor [39]. The results indicate a significant role of the PRDM1 protein in the development of Warthin’s tumors, which may serve as a target for diagnosis and treatment.

Caveolin-1 is a protein that stimulates the process of tumorigenesis but can also play a suppressive role depending on the type of tumor [90,91]. A study by Jaafari-Ashkavandi et al. assessed the expression of Caveolin-1 between benign (15 pleomorphic adenomas) and malignant tumors compared to normal salivary gland tissue. They demonstrated statistically significant overexpression of Caveolin-1 in both benign and malignant tumors compared to the control group, with no differences in expression between benign and malignant tumors. Additionally, for pleomorphic adenomas, they observed an inverse correlation between Caveolin-1 expression and the Ki67 proliferation index. According to the authors, these findings indicate a significant role of Caveolin-1 in the development of salivary gland tumors and its potential use as a diagnostic biomarker [41].

Glypican-3 (GPC-3) is a proteoglycan that regulates cell proliferation, abundantly found in embryonic tissues but sparse in adult tissues [92]. Disruptions in the expression of this molecule may contribute to tumorigenesis [93]. In a study by Tadbir et al., they assessed GPC-3 expression in salivary gland tumors, including 17 benign tumors (pleomorphic adenomas), compared to the normal parenchyma of the surrounding salivary gland [51]. They demonstrated an overexpression of GPC-3 in tissues from salivary gland tumors, including in 11 out of 17 pleomorphic adenomas, compared to healthy tissue; however, this expression was more frequent and more substantial in malignant tumors than in benign ones (64.7% for PA vs. 82.4% for ACC vs. 87.5% for MEC) [51]. The authors suggest using GPC-3 as a target for potentially targeted salivary gland tumor therapy [51].

The RANK receptor and its ligand RANKL are factors involved in bone metabolism [94,95] and have also been widely described in the literature as significant factors in the carcinogenesis of many malignant tumors being detected in the tumor microenvironment [95]. In a study by Aslan et al., they compared the expression of RANK and RANKL between 50 malignant and 38 benign salivary gland tumors [52]. RANK expression was detected in 82% of malignant tumors compared to 34.2% in benign tumors, and in the case of RANKL, 28% vs. 5.3% in favor of malignant tumors. This may indicate the potential of RANK and RANKL as biomarkers that could be used in the differential diagnosis of benign and malignant salivary gland tumors.

Periostin is an extracellular matrix protein that plays structural roles and regulates fibrosis processes [96]. It is also associated with chronic inflammation [97]. Tateda et al. used specific antibodies to study periostin expression in 38 benign parotid gland tumors removed during surgery [65]. An increased expression of this protein was found in 32 out of 38 (84.2%) tissue samples, suggesting its involvement in the pathogenesis of benign salivary gland tumors and its potential use as a diagnostic biomarker [65].

## 5. Conclusions

In summary, the above literature review highlights the significant role of factors related to inflammation and benign salivary gland tumors. Knowledge of the etiopathogenesis of these tumors remains insufficient, and the rich immunological background of these tumors poses challenges in diagnosis. Despite the benign nature of these tumors, surgical treatment carries the risk of potentially significant complications, such as facial nerve paralysis. Changing the approach to diagnosis and treatment could minimize the risk of misdiagnosis, and new potential targeted drugs could serve as alternatives or support surgical treatment. The findings also point to directions for further clinical research, which will be necessary to implement these molecules in clinical practice.

## Figures and Tables

**Table 1:** Characteristic of included studies.

Study	Number of Patients with Benign SGT	Studied Molecule	Effect on Benign Tumor Tissues or Serum
Loy et al., 2005 [20]	21	COX-2	Overexpression/ upregulation
Andreadis et al., 2006 [21]	54	E-cadherin	Strong overexpression
Pantelis et al., 2008 [22]	5	Human beta-defensin 1/2/3	Decreased expression of hBD-1, no significant difference regarding hBD-2 and hBD-3
Tampouris et al., 2011 [23]	20	VEGF-C/VEGF-D/ VEGFR-3 (flt-4)	All 3 strongly expressed in PA, VEGF-C/D moderately expressed or not expressed in other benign tumors flt-4 strongly expressed in all benign tumors
Liu et al., 2012 [24]	72	Chemokine CCL28	Decreased expression
Kehagias et al., 2012 [25]	18	N-cadherin	Expressed in some cases of Warthin’s tumor, not expressed in other benign samples
Carlesimo et al., 2012 [26]	2	TNF-alpha	Not analyzed in the tissue
Jour et al., 2013 [27]	14	Cyclin D1 and p16INK4A	Almost all benign tumors expressed p16 and cyclin D1
Andisheh Tadbir et al., 2013 [28]	31	VEGF	Higher concentration in serum
Aga et al., 2013 [29]	1	IgG4	Serum and tissue levels increased
Ohtomo et al., 2013 [30]	14	SOX-10	Present in all PA, ME, absent in some of WT
Donadio et al., 2013 [31]	36	26 proteins	Characteristic separate chains of proteins for PA and WT
Ianez et al., 2013 [32]	101	CD24/CD44	CD24/CD44 positive by immunochemistry, CD44, also by PCR
Karbanová et al., 2014 [33]	10	Prominin-1 (CD133)	Overexpressed
Reshma et al., 2014 [34]	15	Maspin	Overexpressed
Aga et al., 2014 [35]	37	IgG4	Serum and tissue levels increased in some of WT, mRNA overexpressed in some of WT, not increased/expressed in PA
Faur et al., 2014 [36]	20	VEGF	Moderately positive VEGF expression
He et al., 2014 [37]	3	Phosphatidylocholine	Increased in WT
Andisheh-Tadbir et al., 2014 [38]	15	CD166	Overexpressed
Wang et al., 2015 [39]	40	PRDM1	Pverexpressed
Haghshenas et al., 2015 [40]	19	IL-17-producing lymphocytes and CTLA4+ lymphocytes	Increased concentration in serum
Jaafari-Ashkavandi et al., 2015 [41]	15	Caveolin-1	overexpressed
Fonseca et al., 2015 [42]	120	Semaphorins and neutrophilins	No significant difference vs. control group
Khademi et al., 2016 [43]	50	INF y, IL-4	No significant difference vs. control group
Kim et al., 2016 [44]	8	IgG4	Increased concentration in serum
Haghshenas et al., 2016 [45]	15	Th1, Th2, Tc1, Tc2 lymphocytes	No significant difference vs. control group
Zare et al., 2018 [46]	14	IL-33	Slightly overexpressed
Sowa et al., 2018 [47]	51	Adipocytokines	Serum levels of adiponectin and visfatin elevated, leptin elevated in males, IL-6 no difference
Sowa et al., 2018 [48]	26	Protein oxidation products	Serum level of Total Antioxidant Capacity of Blood Serum and thiol groups decreased, Advanced oxidation protein products increased
Błochowiak et al., 2018 [49]	45	VEGF165b, VEGFR1, VEGFR2 and CD34	No significant difference vs. control group
Tenorio et al., 2018 [50]	38	COX-2, cyclin D1	Underexpressed vs. malignant tumors
Andisheh-Tadbir et al., 2019 [51]	17	Glypican-3 (GPC-3)	Overexpressed
Aslan et al., 2020 [52]	38	RANK, RANKL	Underexpressed vs. malignant tumors
Baneckova et al., 2020 [53]	89	S1009, SOX-10	Expressed in 10% of oncocytic cases of PA/ME, negative in oncocytoma
Da Silva et al., 2020 [54]	51	ALDH-1	Expressed in parenchyma of all benign tumors
Mochizuki et al., 2021 [55]	40	CXCL10, CXCL12, CCL18	WT mostly positive for expression, PA mostly negative
Kobayashi et al., 2021 [56]	64	Th, Tfh	Different for solid-type and cyst-type tumor
Sahin et al., 2022 [57]	185	Systemic Immune-Inflammation Index (SII)	Lower vs. malignant tumors
Haghshenas et al., 2022 [58]	5	MSC cells	Many cell expressed—CD44, CD73, CD90, CD105, and CD166, heat shock protein 70 (Hsp70), keratin, type II cytoskeletal 7 (CK-7),
Laohavisudhi et al., 2022 [59]	13	CD44s, CD44v6, CXCR2, CXCL1, and IL-1β	CD44s, CD44v6, CXCR2—increased in benign tumors
Gaonkar et al., 2022 [60]	15	Endoglin	Higher expression vs. control group, lower vs. malignant tumors
Abbate et al., 2022 [61]	191	NLR, SII, PRL	All markers significantly increased vs. control group
Sowa et al., 2022 [62]	52	Oxidative stress markers	Plasma lipofuscin increased in all benign tumors, Cu-Zn SOD decreased in WT
Jabbar et al., 2023 [63]	30	VEGF	Overexpression
Abbate et al., 2023 [64]	140	Inflammatory biomarkers SII, SIRI, PLR, and NLR	SIRI showed highest accuracy in determining malignancy, decreased vs. malignant tumors
Tateda et al., 2024 [65]	38	Periostin	Overexpression in 32 out of 38 benign tumors

Abbreviations: PA—pleomorphic adenoma, WT—Warthin’s tumor, ME—myoepithelioma, VEGF—vascular endothelial growth factor, MSC—mesenchymal stem cells, NLR—neutrophil to lymphocyte ratio, SII—systemic immune-inflammation index, PLR—platelet to lymphocyte ratio, SIRI—systemic inflammation response index.

## Data Availability

Data are contained within the article.

## Abbreviations

WT—Warthin’s tumor, PA—pleomorphic adenoma, TME—tumor microenviroment, ME—myoepithelioma.
